# Elevated Intracranial Pressure Diagnosis with Emergency Department Bedside Ocular Ultrasound

**DOI:** 10.1155/2015/385970

**Published:** 2015-10-26

**Authors:** D. Amin, T. McCormick, T. Mailhot

**Affiliations:** Department of Emergency Medicine, Los Angeles County-University of Southern California, Los Angeles, CA 90033, USA

## Abstract

Bedside sonographic measurement of optic nerve sheath diameter can aid in the diagnosis of elevated intracranial pressure in the emergency department. This case report describes a 21-year-old female presenting with 4 months of mild headache and 2 weeks of recurrent, transient binocular vision loss. Though limited by patient discomfort, fundoscopic examination suggested the presence of blurred optic disc margins. Bedside ocular ultrasound (BOUS) revealed wide optic nerve sheath diameters and bulging optic discs bilaterally. Lumbar puncture demonstrated a cerebrospinal fluid (CSF) opening pressure of 54 cm H_2_O supporting the suspected diagnosis of idiopathic intracranial hypertension. Accurate fundoscopy can be vital to the appropriate diagnosis and treatment of patients with suspected elevated intracranial pressure, but it is often technically difficult or poorly tolerated by the photophobic patient. BOUS is a quick and easily learned tool to supplement the emergency physician's fundoscopic examination and help identify patients with elevated intracranial pressure.

## 1. Introduction

Visual complaints are common in the emergency department (ED), and many ocular emergencies are time sensitive [1]. However, the proper diagnosis and management of ocular emergencies by emergency physicians can be limited by lack of sophisticated tools and training. Ocular ultrasound is considered to be a core indication for emergency ultrasound by the American College of Emergency Physicians [2], and bedside ocular ultrasound (BOUS) is becoming a vital aspect of the eye exam for patients with eye complaints. BOUS is sensitive for the diagnosis of retinal detachment (RD) and may have a role in excluding RD in patients presenting to the ED with “floaters,” “flashes,” and vision loss [3–5]. Another promising application of BOUS is the assessment of optic nerve sheath diameter in patients with suspected intracranial hypertension. A prospective study of ICU patients with intracranial pressure monitors in place showed a strong correlation between an optic nerve sheath diameter of more than 5 mm and intracranial pressure greater than 20 cm H_2_O [5]. We describe a case of a young female presenting with long-standing headache and vision loss. BOUS revealed widened and bulging optic nerve sheaths bilaterally, and she was subsequently diagnosed with idiopathic intracranial hypertension.

## 2. Case Report 

A 21-year-old female with no past medical history presented to the ED complaining of 2 weeks of recurrent, transient binocular vision loss. The episodes lasted approximately 30 seconds and were increasing in frequency, occurring 6 times on the day of presentation. In between episodes, the patient reported normal vision. On further questioning, the patient reported a mild throbbing headache for 4 months. The pain started in the upper neck and radiated to the top of the head and bilateral shoulders like an electric shock; it was worse with bending forward. She denied photophobia, nausea, vomiting, fevers, chills, neck stiffness, numbness, or weakness. She was taking no medications or oral contraceptives. She had no personal or family history of venous thromboembolism. On physical examination her temperature was 37.2°C, heart rate 69 beats/min, respiration rate 16 breaths/min, and blood pressure 105/69 mmHg. Finger-stick glucose was 84 mg/dL. Her corrected visual acuity was 20/25 in her right eye and 20/25 in her left eye. Her extraocular motions were intact and pupils were equally round and reactive to light and accommodation. Visual fields were intact. Fundoscopic examination was poorly tolerated due to patient discomfort; however, there appeared to be optic disc edema bilaterally. She had no nuchal rigidity, and Kernig and Brudzinski signs were negative. The remainder of her HEENT, cardiovascular, pulmonary, abdominal, and neurologic examinations were normal.

**Figure 1:**
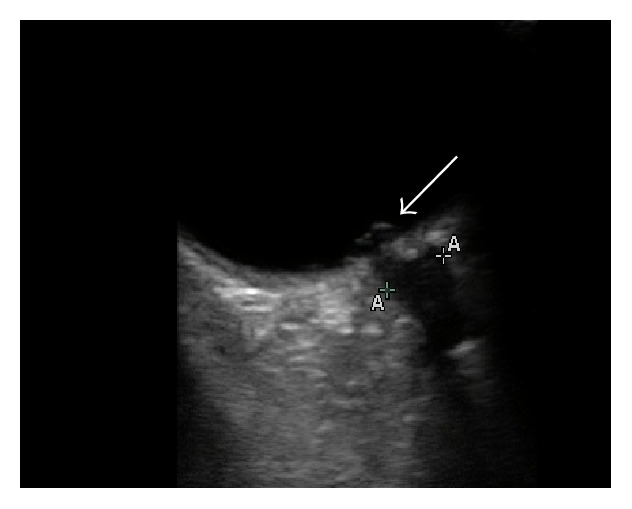
Bedside ocular ultrasound of the right eye demonstrating bulging of the optic nerve cup, indicative of papilledema.

**Figure 2:**
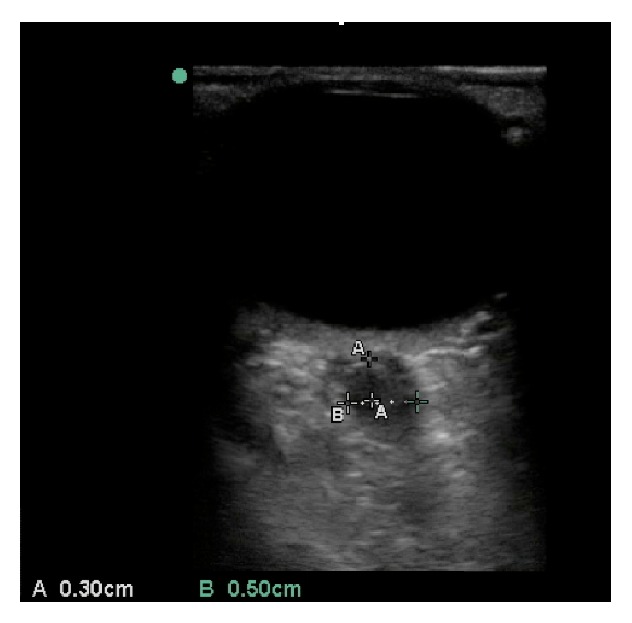
Bedside ocular ultrasound of the patient's left eye demonstrating optic nerve sheath diameter (ONSD) measurement. The measurement of the ONSD is taken 3 mm posterior to the globe and occurs perpendicular to the long axis of the optic nerve.

**Figure 3:**
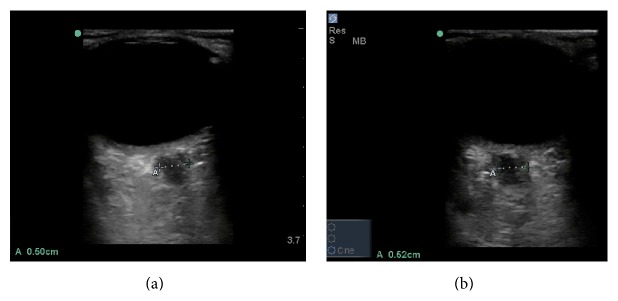
Bedside ocular ultrasound of left (a) and right (b) eyes, showing measurement of optic nerve sheath diameter. Measurements are 5 mm and 5.2 mm, respectively.

BOUS was performed using a 13–6 MHz linear array transducer (SonoSite M-Turbo, Bothell, WA). It showed a hyperechoic prominence protruding into the posterior chamber at the location of the optic nerve (Figure 1). The optic nerve sheath diameter measured 3 mm posterior to the globe was 5.0 mm in the right eye and 5.2 mm in the left eye (Figures 2 and 3). Noncontrast computed tomography of the brain was unremarkable. Opening cerebrospinal fluid pressure during lumbar puncture was 540 mm H_2_O. The patient's headache improved significantly after the lumbar puncture. Neurology was consulted and recommended starting the patient on acetazolamide. MRI without contrast was normal and her symptoms were well controlled on acetazolamide 3 months later.

## 3. Discussion

Physical examination of the eye is significantly limited in patients who are photophobic, intubated, or unable to follow commands, and accurate detection of papilledema can be technically difficult even in a cooperative patient. Furthermore, papilledema may be delayed after elevations in intracranial pressure (ICP) by several hours to days [7]. A small prospective study of head injured patients with suspected intracranial injury found that an optic nerve sheath diameter of >5 mm was 100% sensitive (95% CI 68 to 100%) for the presence of elevated ICP [8]. These findings suggest that BOUS may have an even more important role in detecting acute increased ICP than the fundoscopic exam. However, the possibility of elevated ICP without papilledema or increased ONSD should be considered in the appropriate clinical context.

Anatomically, the optic nerve is a part of the central nervous system, which is surrounded by the dura mater, the subarachnoid space, and cerebrospinal fluid; therefore, any change in ICP affects the optic nerve sheath, changing its diameter. Helmke and Hansen first described the use of ocular ultrasound to detect increased ICP in cadavers in 1996 [9]. Blaivas et al. described close correlation between optic nerve sheath dilation and CT evidence of elevated ICP in ED patients with head trauma and spontaneous intracranial hemorrhage [10]. The relationship between ONSD and ICP has been well established and this case report contributes to the rising body of evidence that elevated ICP can be rapidly and noninvasively detected using BOUS.

Bulging of the optic nerve into the posterior chamber was also seen on BOUS in this patient. While ONSD has been studied in several prospective trials, data on optic disc bulging into the posterior chamber is limited to case reports [6, 11]. This finding may be a more direct sonographic correlate to the physical exam finding of papilledema than ONSD, but further research is needed to determine its significance.

While elevated ICP may be the most concerning and time sensitive cause of a widened ONSD and optic disc bulging for the emergency physician, inflammatory, infectious, and ischemic diseases of the optic nerve can result in similar BOUS findings [6] and should be considered in the appropriate clinic setting.
